# Effect of Star-like Polymer on Mechanical Properties of Novel Basalt Fibre-Reinforced Composite with Bio-Based Matrix

**DOI:** 10.3390/polym16202909

**Published:** 2024-10-16

**Authors:** Rochele Pinto, Tatjana Glaskova-Kuzmina, Kristina Zukiene, Gediminas Monastyreckis, Marie Novakova, Vladimir Spacek, Andrejs Kovalovs, Andrey Aniskevich, Daiva Zeleniakiene

**Affiliations:** 1 Department of Mechanical Engineering, Kaunas University of Technology, Studentu St. 56, 51424 Kaunas, Lithuania; tatjana.glaskova-kuzmina@ktu.lt (T.G.-K.); gediminas.monastyreckis@ktu.lt (G.M.); daiva.zeleniakiene@ktu.lt (D.Z.); 2 Department of Production Engineering, Kaunas University of Technology, Studentu St. 56, 51424 Kaunas, Lithuania; kristina.zukiene@ktu.lt; 3 SYNPO, S. K. Neumanna 1316, 530 02 Pardubice, Czech Republic; marie.novakova@synpo.cz (M.N.); vladimir.spacek@synpo.cz (V.S.); 4 Institute of Materials and Structures, Riga Technical University, Kipsalas 6a, LV-1048 Riga, Latvia; andrejs.kovalovs@rtu.lv; 5 Institute for Mechanics of Materials, University of Latvia, Jelgavas 3, LV-1004 Riga, Latvia; andrey.aniskevich@pmi.lu.lv

**Keywords:** star-like polymer, basalt fibre, bio-based matrix, mechanical properties

## Abstract

This study is aimed at developing a fibre-reinforced polymer composite with a high bio-based content and to investigate its mechanical properties. A novel basalt fibre-reinforced polymer (BFRP) composite with bio-based matrix modified with different contents of star-like n-butyl methacrylate (*n*-BMA) block glycidyl methacrylate (GMA) copolymer has been developed. *n*-BMA blocks have flexible butyl units, while the epoxide group of GMA makes it miscible with the epoxy resin and is involved in the crosslinking network. The effect of the star-like polymer on the rheological behaviour of the epoxy was studied. The viscosity of the epoxy increased with increase in star-like polymer content. Tensile tests showed no noteworthy influence of star-like polymer on tensile properties. The addition of 0.5 wt.% star-like polymer increased the glass transition temperature by 8.2 °C. Mode-I interlaminar fracture toughness and low-velocity impact tests were performed on star-like polymer-modified BFRP laminates, where interfacial adhesion and impact energy capabilities were observed. Interlaminar fracture toughness improved by 45% and energy absorption capability increased threefold for BFRP laminates modified with 1 wt.% of star-like polymer when compared to unmodified BFRP laminates. This improvement could be attributed to the increase in ductility of the matrix on the addition of the star-like polymer, increasing resistance to impact and damage. Furthermore, scanning electron microscopy confirmed that with increase in star-like polymer content, the interfacial adhesion between the matrix and fibres improves.

## 1. Introduction

Fibre-reinforced plastics (FRPs) are widely used in applications that require lightweight but resilient structures. They serve as a great alternative to metal counterparts due to their high strength-to-weight ratio and flexibility in modification of design to fabricate complex structures. From automotive industries to construction applications, gradually, heavy metal components are being replaced by carbon or glass FRP composites. In addition to glass and carbon, basalt fibres are increasingly being used for their high strength and durability. While glass and basalt fibres have similar manufacturing processes which involve homogenous melting, extrusion, formation of fibre, and winding, both processes look different internally. The production of glass fibres involves feeding of different raw materials, mainly borosilicates, while basalt fibres have a relatively less complex method of production with crushed basalt rocks as the only raw materials required [1]. Carbon fibres are produced from majorly polymer precursors known as polyacrylonitrile along with rayon with complex processes such as spinning, stabilizing, carbonization, surface treatment, and sizing [2]. Production of both glass and carbon fibres poses toxicity to human health as inhalation of glass micro-fibre can damage the lungs [3] and carbon fibre dust is considered as a carcinogen [4], while the production of basalt fibre has been proved harmless to human health [5]. Additionally, conventional composites comprising of glass or carbon fibres have poor end-of-life recyclability. While various methods have been devised to tackle this issue such as recycling by pyrolysis [6], solvolysis [7], microwave, and thermolysis [8], overall, the production of these materials causes a high carbon footprint. With sustainability in mind, the research on natural fibres has exponentially increased. Flax [9], hemp [10], jute [11], etc. are such fibres that are replacing conventional FRP materials due to their low cost, sustainable production, and end-of-life recyclability [12]. However, most plant- or animal-based natural fibre reinforcements perform poorly due to their weak thermal stability [13] and durability, low mechanical properties [14], and high hydrophilicity [15]. Basalt fibre is an inorganic, synthetic fibre with a high strength-to-weight ratio, excellent corrosion resistance [16], and thermal stability [17]. These mineral-based fibres could bridge the gap between the poor mechanical properties of other natural fibres and the copious amounts of waste produced from conventional FRP material. While offering such attractive attributes, basalt fibres have their drawbacks. The chemical composition of the fibres largely depends on the region from where the rocks are sourced and therefore no consistency in production standards is maintained [18]. Due to their relatively simple method of production with no additives, the surface of the fibres is in turn smooth, causing poor adhesion between the fibre and matrix. The use of fillers such as MXenes, carbon nanotubes, graphene, polyhedral oligomeric silsesquioxane, star polymers, etc. is implemented to improve adhesion between the matrix and basalt fibres [19,20,21,22,23]. Star-like polymers, generally consisting of functional, multiple arm-like structures radiating from a central core producing highly compact and branched-out structures [24], can also be used to enhance their properties. These polymers have been shown to improve the interfacial adhesion in carbon fibre-reinforced polymer composites (CFRPs) and consequently their mechanical properties [25].

While the use of natural fibres can lower the burden on relying on conventional reinforcements, the use of crude oil-based epoxy and curing agents still poses a hurdle in sustainability advancements. While thermoplastic materials are preferred over thermosetting due to their recyclability, they fall short in high-strength applications [26]. Starting materials such as bisphenol A [27] and isophorone diamine [28] for epoxy and curing agents, respectively, are considered highly toxic and dangerous to human health and the environment. On the basis of previously conducted life-cycle assessments, it was noted that higher energy savings were noted for materials with precursors derived from bio-based sources when compared to precursors based on petrochemical products [29]. Bio-based materials that look to naturally occurring products as starting materials contribute to an overall much ‘greener’ composite when used in conjunction with natural or plant-based fibres. Soybean oil [30], linseed oil [31], plant waste [32], and cardanol [33] as precursors are some of the ways manufacturers of thermosetting resins and curing agents are implementing sustainability in production. It has been observed that these materials have provided a stiff competition to conventionally-produced thermosetting materials [34]. Additionally, studies show that basalt fibres have poor adhesion to thermoplastic resins such as polypropylene and in turn show degradation in mechanical properties [5,35].

Investigations on the impact behaviour of BFRP composites have been extensively performed due to their excellent impact energy absorption capabilities. BFRP composites show higher impact performance, with higher levels of impact energy absorbed than CFRP composites due to their high stiffness and ductility [36]. The same case was observed between E-glass and basalt FRPs [37]. In hybrid carbon/basalt fibre laminates with different stacking sequences, basalt fibre plies were shown to resist penetration while carbon fibre plies were completely penetrated [38]. Furthermore, it was observed that hybridisation of flax fibre laminates with basalt fibres could potentially improve the Mode-I interlaminar fracture toughness and moisture resistance in natural fibre composites [39]. The introduction of a basalt fibre veil at the mid-plane of the laminate during fabrication of a BFRP composite has been shown to increase the interlaminar fracture toughness twofold [40]. In a comparative study between natural and conventional FRPs, BFRPs showed comparable mechanical performance and even better Mode-I and Mode-II fracture toughness properties [41].

The aim of this study is to develop a high bio-based content, novel basalt fibre-reinforced polymer composite with improved interfacial adhesion between the matrix and fibre, and through experimental testing, demonstrate its excellent mechanical properties. This composite was inspired by a previously conducted study [25], where star-like polymers were used to improve mechanical properties of CFRP composites. In this study, we observed significant matrix bridging occurring due to increased ductility of the matrix within the laminate. Therefore, a bio-based matrix modified with different contents of star-like *n*-butyl methacrylate (*n*-BMA) block glycidyl methacrylate (GMA) copolymer and reinforced with basalt fibres has been developed. The matrix comprising bio-content of ~33% is modified with star-like polymer and tested for improvement in rheological, tensile, and visco-elastic properties and change in glass transition temperatures. The modified bio-based matrix is then reinforced with basalt fibres and tested for enhancement in mechanical properties. Low-velocity impact tests were performed to observe the energy absorbing capabilities of the composite and Mode-I interlaminar fracture toughness tests to examine the interfacial adhesion between the modified matrix and fibre was performed. Scanning electron microscopy was conducted on post-fractured specimens to visually inspect the various damage modes.

## 2. Materials and Methods

### 2.1. Materials

For the synthesis of star-like polymer, tetrabutyl ammonium acetate, methyl trimethylsilyl dimethylketene acetal, cross-linker ethylene glycol dimethacrylate (EGDMA), and monomers BMA and GMA were sourced from Sigma-Aldrich (St. Louis, MI, USA). Tetrahydrofuran (THF) of 99.8% purity was obtained from VWR Chemicals (Radnor, PA, USA).

**Figure 1:**
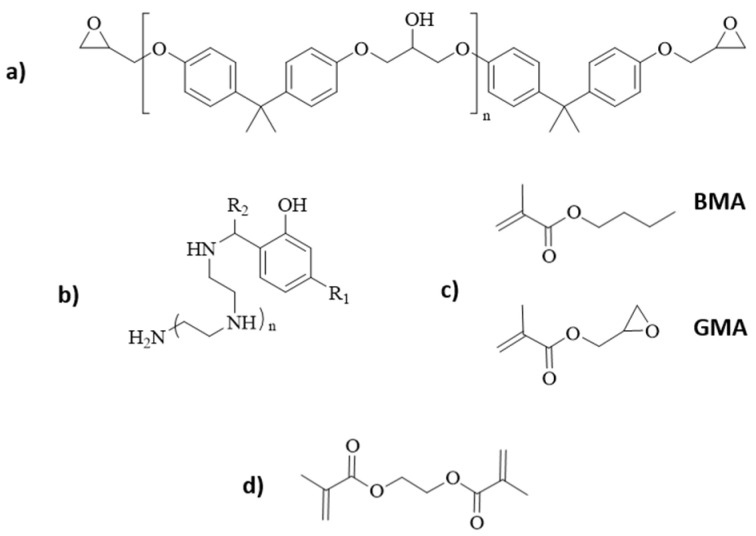
Chemical structures of: (**a**) DGEBA; (**b**) phenalkamine; (**c**) BMA and GMA; and (**d**) EGDMA.

For the matrix, bio-based diglycidyl ether of bisphenol A epoxy SR Greenpoxy 33 (Sicomin, Châteauneuf-les-Martigues, France) with carbon content (~35%) sourced from plant origins and solvent-free phenalkamine epoxy curing agent LITE 2401 (Gent, Cardolite, Belgium) sourced from cashew nutshell liquid technology with bio-content of ~33% were chosen. LITE 2401 was preferred so as to not lower the overall bio-content of the mixture. Chemical structures of materials used are presented in Figure 1.

Twill-woven basalt fabric (Basaltex, Wevelgem, Belgium) with density of 220 g/m^2^ was used as reinforcement in BFRP laminates for low-velocity impact tests. Unidirectional basalt fabric BAS UNI 350 (Basaltex, Belgium) of density 416 g/m^2^ was used as reinforcement in BFRP laminates for interlaminar toughness tests.

### 2.2. Synthesis of Star-like Polymer

Star-like *n*-BMA block GMA copolymer was synthesized using the ‘arm-first’ group transfer polymerisation method [25]. In a three-neck round bottom flask, tetrabutyl ammonium acetate, methyl trimethylsilyl dimethylketene acetal, and THF used as catalyst, initiator, and solvent, respectively, were added. BMA and GMA monomers were slowly added dropwise. The mixture was stirred for 25 min after the addition of the monomers after which the cross-linker EGDMA was added.

**Figure 2:**
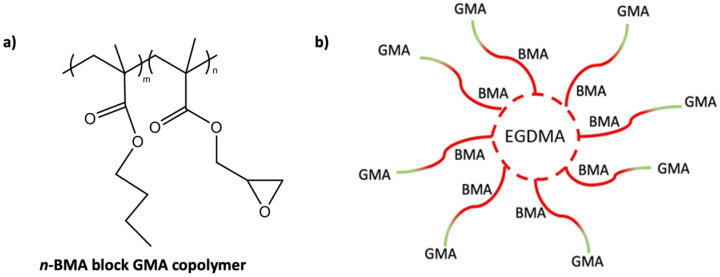
Star-like polymer structure: (**a**) chemical structure of star-like polymer arms; and (**b**) schematic diagram for star-like polymer structure.

After the polymerisation, the solid star-like polymer was stored in 48% THF. The molecular weights of the core and arms are ~87,000 and ~3000 g/mol, respectively, and were determined using gel permeation chromatography. The arms of the star-like polymer consist of ~28 units of *n*-BMA and ~2 units of GMA. The core is formed by partially cross-linked arms by copolymerisation, giving the appearance of a star and therefore called ‘star-like polymer’ with additional free arms in solution (Figure 2).

### 2.3. Material Preparation

Star-like polymer was added to the epoxy resin at different contents ranging from 0.25 to 7 wt.% to investigate its influence from smaller to higher contents. The mixtures were stirred for 24 h at 60 °C until fully combined. The curing agent was added according to the equivalent weight of the epoxy after adding additives and degassed until all bubbles escaped.

**Figure 3:**
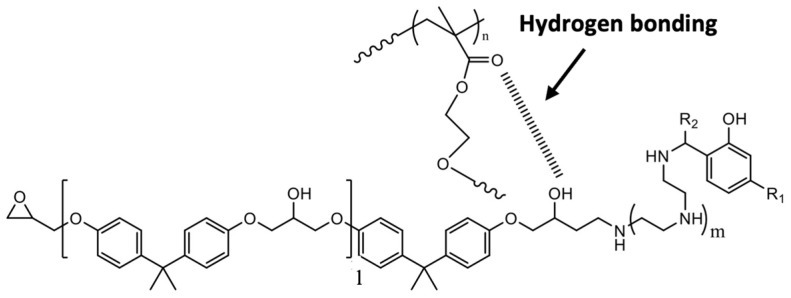
Schematic representation of possible hydrogen bonding interaction between epoxy and *n*-BMA block GMA copolymer.

The modified epoxy was cured at room temperature for 24 h and post-cured in the oven for 2 h at 80 °C and 2 h at 120 °C. The BFRP laminates were fabricated using the hand-layup technique, vacuum bagged overnight, and the same curing cycle was followed. During curing, both the epoxy and the GMA block of star-like polymer react with the phenalkamine through epoxy group ring-opening. An interaction between the star polymer and the epoxy is also possible due to the hydrogen bonding of the hydroxyl groups of the epoxy to the carbonyl groups of the *n*-BMA block GMA copolymer (Figure 3) [42].

### 2.4. Fabrication of Specimens

For the tensile tests, the specimens were moulded according to ASTM D638 Type-II [43]. The bio-based matrix was modified with 0.25, 0.5, 1, 5, and 7 wt.% of star-like polymer and compared with neat epoxy. For dynamic mechanical analysis (DMA) tests, three different contents of star-like polymer were chosen to modify the bio-based matrix: 0.25, 0.5, and 1 wt.% as higher contents of star-like polymer showed high viscosity, lower stiffness, and presence of bubbles which could lead to early failure. The specimens were moulded according to ASTM D4065-20 [44] with dimensions of 30 × 3 × 1 mm. At least five specimens were fabricated and tested.

The specimens for Mode-I interlaminar fracture toughness tests on BFRP modified with 0.5 and 1 wt.% of star-like polymer were prepared according to ASTM D5528 [45] and had dimensions 125 × 25 × 3 mm. To obtain a composite plate of 3 mm thickness, 12 sheets of unidirectional fibre fabric of 200 × 150 mm were needed. Each layer of the fabric was laid in a special lodge and impregnated with epoxy resin. After laying the first six layers, a fluoroplastic separation film was placed from one edge of the plate following which the remaining layers of the laminate were laid. A lodge with fibre fabric was placed in a hydraulic press under 20 kPa load for 24 h at room temperature. Then, the laminate plates were cut, and the above-mentioned curing cycle was followed. At least five double cantilever beam (DCB) specimens were fabricated and tested.

**Figure 4:**
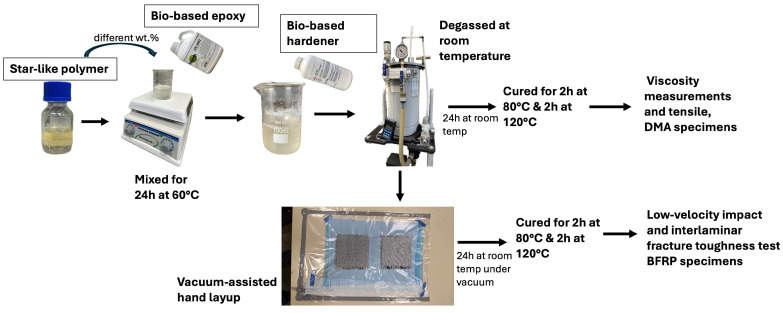
Materials’ and specimens’ preparation process.

Low-velocity instrumented puncture tests specimens were fabricated according to ISO 6603-2 [46] with dimensions of 60 × 60 × 1 mm. Two different star-like polymer contents, 0.5 and 1 wt.%, were chosen to be reinforced with basalt fibres. The laminates were fabricated by hand-layup and five layers of basalt fibre were used to achieve a thickness of 1 mm. The specimens were vacuum bagged for 12 h and the above-mentioned curing cycle was followed. At least five specimens were fabricated and tested. Only two contents were chosen for the modification of BFRP laminates as significant results were not seen on the addition of 0.25 wt.% in the bio-based matrix. The overall process of materials’ and specimens’ preparation is presented in Figure 4.

### 2.5. Materials Testing

Rheological behaviour of modified epoxy was investigated by viscosity measurements using a Brookfield DVII+ Pro Viscometer (Brookfield Engineering, Middleboro, MA, USA). Tests were performed at room temperature (23 °C) with five repetitions to take repeatability into consideration. An LV5 spindle was used to measure the viscosity.

Tensile tests on star-like polymer-modified bio-based epoxy were performed according to the ISO-527 standard [47]. The tests were conducted on an H10 KT universal column testing machine (Tinius Olsen, Redhill, UK) with a crosshead speed of 2 mm/min. All tests were performed at room temperature. The Young’s modulus was calculated within 0.05–0.25% strain rates according to the standard. For each mixture, at least six specimens were tested and average values were calculated.

DMA was implemented to study the effect of star-polymer content on the matrix’s visco-elastic properties. DMA tests were conducted in tension mode with a force of 2 N using a Mettler Toledo (Columbus, OH, USA) DMA/SDTA861 instrument to estimate the thermomechanical properties. All tests were performed at a frequency of 1 Hz, temperature range between 30–180 °C, and at a heating rate of 3 °C /min to ensure slow heating.

Mode-I interlaminar fracture toughness tests were carried out according to ASTM D5528-01 [45] using a Zwick 2.5 kN (Zwick Roell Group, Ulm, Germany) machine at a crosshead speed of 1 mm/min at room temperature and a Canon EOS40D (Canon, Tokyo, Japan) to visualise the crack propagation analysis until failure at every three seconds. To estimate the length of delamination, ImageJ 1.38x software was used. To calculate the energy strain release rate at the initiation of delamination, a linear elastic behaviour is assumed owing to the relatively small damage area when compared the thickness of the specimens. To estimate Mode-I interlaminar fracture toughness, the modified beam theory equation [48] (Equation (1)) was used to correct the rotation at the initiation of delamination by visualising the DCB with a marginally longer delamination, *a* + Δ, where Δ is the absolute value of the intercept of the least-squares graph of the cube root of compliance *C*^1/3^ vs. delamination length and *a* indicates the delamination length of the specimen:

(1) $$G_{I}=\frac{3P\delta }{2b\left(a+\left|\Delta \right|\right)}$$

where *P* represents the load, *δ* denotes the displacement at the load point, *b* stands for the width of the specimen. The compliance *C* is the ratio of *δ/P.*

To incorporate the effect of the loading blocks, two correction parameters are suggested [45]: a parameter *F*, which adjusts for the shortening of the moment arm and the inclination of the blocks, and a parameter *N*, which compensates for the stiffening effect of the blocks on the specimen:

(2) $$F=1-\frac{3}{10}{\left(\frac{\delta }{a}\right)}^{2}-\frac{3}{2}\left(\frac{\delta t}{a^{2}}\right)$$

(3) $$N=1-{\left(\frac{L^{\prime }}{a}\right)}^{3}-\frac{9}{8}\left[1-{\left(\frac{L^{\prime }}{a}\right)}^{2}\right]\left(\frac{\delta t}{a^{2}}\right)-\frac{9}{35}{\left(\frac{\delta t}{a^{2}}\right)}^{2}$$

where $L^{\prime }$ and *t* are the dimensions of the blocks.

Then, the modified equation for interlaminar fracture toughness is presented as:

(4) $$G_{I}=\frac{3P\delta }{2b\left(a+\left|\Delta \right|\right)}\cdot \left(\frac{F}{N}\right)$$

Low-velocity impact tests were carried out using the Instron Dynatup 9250 HV Impact Tower test machine (Norwood, MA, USA) at an impact energy of 50 J and velocity of 3.5 m/s. The hemispherical impactor with diameter 25.4 mm was connected with a force transducer of 16 kN capacity to measure the impact force during the test. The specimens were clamped between two steel plates with a circular hole of diameter 40 mm. The impactor was dropped from a height of 1 m.

**Figure 5:**
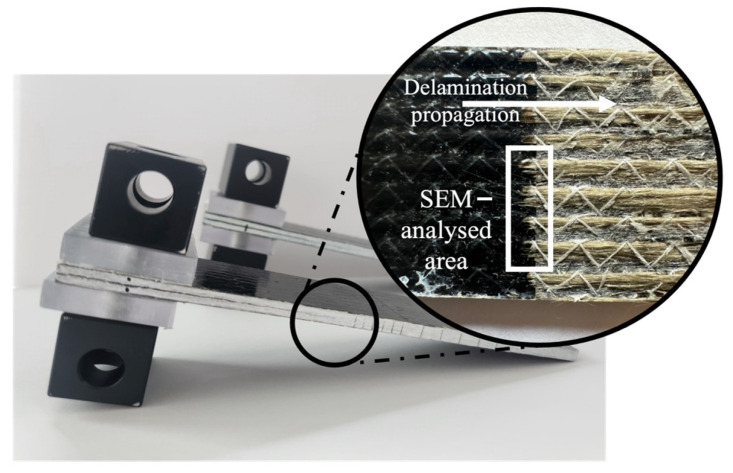
Image of interface depicting delamination initiation and SEM-analysed area.

Scanning electron microscopy (SEM) (S-3400N, Hitachi, Tokyo, Japan) was performed using a BRUKER Quantax EDS detector in high vacuum mode on the BFRP laminates from the interlaminar fracture toughness test. A section of the interface where delamination initiates was chosen (Figure 5) to visualise the failure behaviour.

## 3. Results and Discussion

### 3.1. Effect of Star-like Polymer Content on Viscosity of Bio-Based Epoxy

**Figure 6:**
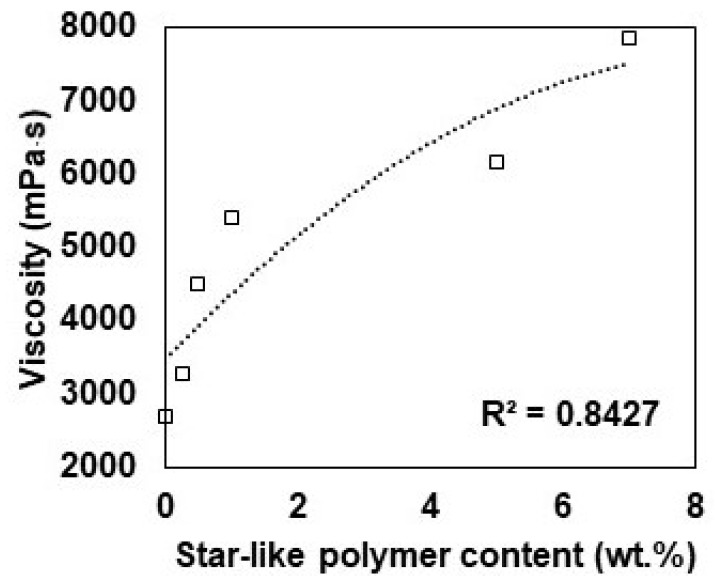
Dependency of viscosity on star-like polymer content.

The rheological properties of the matrix are crucial to understand its influence on its processability, manufacturing, effect on mechanical properties, and wetting ability in FRP composites. As can be seen in Figure 6, the viscosity increased drastically with increasing star-like polymer content. Adding 0.5 wt.% of star-like polymer, the viscosity increases 1.5 times more than neat epoxy. With star-like polymer content above 1 wt.%, the viscosity increases acutely, which decreases the possibility of producing high quality specimens. Increased viscosity in resin reduces the wetting ability of the fibre by the epoxy, hence unsuccessful distribution of the stress through the laminate. The increase in viscosity can be explained in two different ways: first, star-like polymers, due to their non-linear structure, cannot elongate and align in the shear direction and are therefore more shear-stable than their linear counterparts. Second, the star-like polymer has a high molecular weight when compared with epoxy; therefore, adding a small content of star-like polymer increases the overall molecular weight of mixtures.

### 3.2. Tensile Properties of Star-like Polymer-Modified Bio-Based Matrix

**Figure 7:**
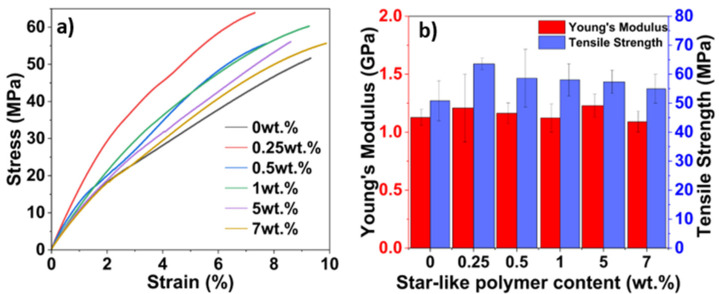
Tensile test results: (**a**) stress-strain curves; (**b**) tensile strength and modulus of the bio-based matrix with different contents of star-like polymer.

To investigate the influence of star-like polymer on the bio-based matrix, tensile tests were performed (Figure 7). Stress-strain curves of the bio-based matrix at five different contents of star-like polymer are presented in Figure 7a. The specimens exhibited increasing elongation for the same range of stress values on the addition of star-like polymer. With star-like polymer content above 1 wt.%, larger elongations with lower stiffness were observed showing the increase in ductility of the matrix.

As can be seen from Figure 7b, the addition of 0.25 wt.% of star-like polymer results in a 24 % improvement in ultimate tensile strength when compared to neat epoxy. This could be a result of the presence of GMA blocks, which are involved in the cross-linking network. This is also confirmed by the lowering in elongation, indicating that the addition of 0.25 wt.% of star-like polymer causes brittleness in the matrix (Figure 7a). As the content of star-like polymer continues to increase up to 1 wt.%, the tensile strength slightly decreases and elongation increases indicating that the ductility of the epoxy increases with the star-like polymer, which could be attributed to the flexible butyl units of BMA blocks. The Young’s modulus of the mixtures remains fairly constant for all investigated cases.

### 3.3. Visco-Elastic Properties of Star-like Polymer-Modified Bio-Based Matrix

**Figure 8:**
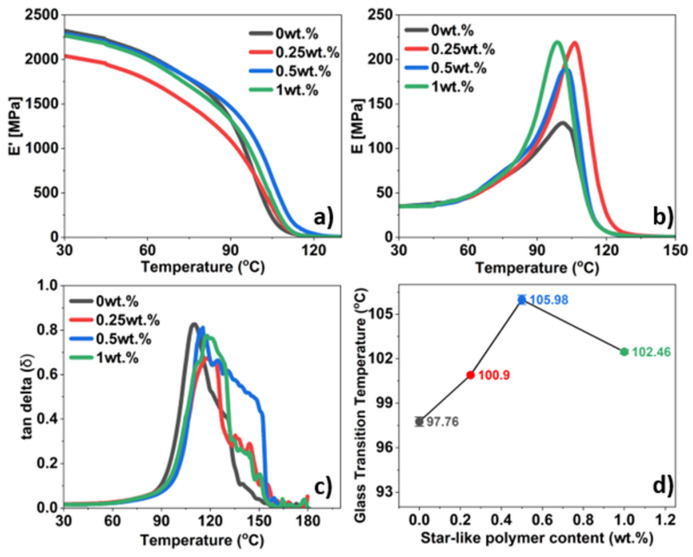
DMA results: (**a**) storage modulus; (**b**) loss modulus; (**c**) damping factor; (**d**) glass transition temperatures of the bio-based matrix with different contents of star-like polymer.

To better understand the visco-elastic properties in response to oscillatory forces, thermomechanical effect, and change in the glass temperature on its inclusion, DMA was performed on different contents of star-like polymer-modified bio-based epoxy. The glass transition temperature (*T_g_*) was determined, based on the temperature dependence of the storage modulus (Figure 8a), identified at the inflection point and shown in Figure 8d as a function of star-like polymer content [49,50]. The changes in *T_g_*, storage modulus, and loss modulus were analysed in response to the stress applied as shown in Figure 8.

The storage modulus (*E*′) of a material corresponds to the amount of energy stored elastically. The storage modulus is seen to decrease with addition of 0.25 and 1 wt.% when compared to neat epoxy but have some reinforcing effect at 0.5 wt.% of star-like polymer (Figure 8a). The steep decrease in the storage modulus is a sign of the possible maximum working temperature for the mixtures. The loss modulus (*E*″) of a material is associated with the energy loss, dissipated in the form of heat as the material turns viscous. High values of loss modulus correlate to viscous behaviour hence increased damping properties [51]. While 0.25 and 1 wt.% do not show high values of loss modulus, the addition of 0.5 wt.% not only has a slightly higher loss modulus (Figure 8b) but also signifies that the *T_g_* has increased by 8.2 °C (Figure 8d). The *T_g_* is an indicator of the maximum working temperature after which the polymer matrix suffers reduced stiffness and thereby may affect the matrix-dominant properties in a laminate.

The damping factor is the ratio of loss modulus to the storage modulus. The curve is stable until 90 °C after which a peak is achieved around the *T_g_* (Figure 8c). The area under the damping factor curve gives an insight into polymer interactions and its mobility in the mixture, and thereby its damping capabilities. With increase in content of star-like polymer, an increase in the area under the damping factor curve is observed. The cross-link density has a significant influence on the local molecular stiffness, which is not apparent in the macroscopically averaged stiffness of the material [52]. Therefore, the heterogeneity in crosslinking is very likely to be enhanced for filler-modified thermosets. Moreover, the emergence of the multiple peaks can be also attributed to the presence of an interphase between the matrix and star-like polymer, exhibiting modified relaxation properties compared to the unmodified bio-based epoxy [53,54].

### 3.4. Interlaminar Fracture Toughness of BFRP Laminates

In the DCB test, as the delamination initiates from the insert, the resistance-type fracture behaviour typically develops where the calculated Mode-I interlaminar fracture toughness *G*_Ic,_ increases monotonically and stabilizes with further delamination growth. The fracture toughness is assessed by analysing the delamination resistance curve (*G*_Ic_ versus *a*) obtained from DCB tests, specifically at the point when deviation from linearity occurs in the load-displacement curve.

**Figure 9:**
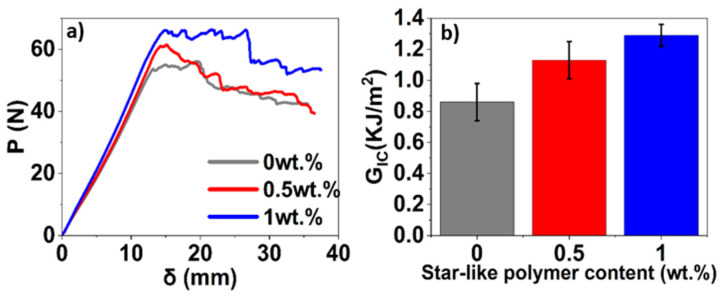
Mode-I interlaminar fracture toughness test results: (**a**) load-COD curves; (**b**) critical energy release rates for different contents of star-like polymer.

Representative load-crack opening displacement (COD) curves obtained for BFRP impregnated with the epoxy with different star-like polymer content are provided in Figure 9a. A rather significant deviation among the data was observed, which is typical for such tests. The fracture toughness evaluated by Equation (4) for BFRP impregnated with the epoxy is shown in Figure 9b.

A significant enhancement in the interlaminar fracture toughness of BFRP is observed due to the presence of star-like polymer. In the load-COD curves (Figure 9a), a stable crack propagation is observed after the onset of crack initiation in all DCB specimens. However, the presence of the star-like polymer increased the onset of crack initiation showing improvement in resistance of the modified BFRP laminates to crack initiation and propagation. This improvement is also evident in the increase in the energy release rate from 0.92 kJ/m^2^ for neat epoxy to a peak value of 1.33 kJ/m^2^ for BFRP containing 1 wt.% of star-like polymer (Figure 9b), a maximal increase of 44.57%. The presence of star-like polymer improves the resistance to delamination and crack propagation within the laminate by improving the adhesion between the matrix and fibre. The addition of star-like polymer improves the ductility of the matrix, causing matrix bridging and possibly some crack arresting phenomenon by micro-cracking in matrix or fibre breakage. Increasing strain energy release rates can be attributed to higher fibre-bridging and cohesive failure occurring at the wake of delamination [55]. Further increasing the fraction of star-like polymer could potentially yield more significant enhancements in the interlaminar fracture toughness of BFRP. However, it is worth noting that increasing the content of star-like polymer increases the epoxy resin’s viscosity, adversely affecting the fabrication of composite laminates. A more elaborate visualisation of the damage morphology of the unmodified and modified BFRP laminates’ post-interlaminar fracture toughness tests using SEM is presented in Section 3.5.

### 3.5. Low-Velocity Impact Properties of BFRP Laminates

**Figure 10:**
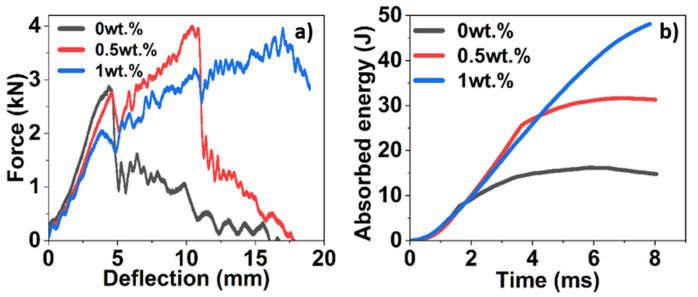
Low-velocity impact tests: (**a**) contact-force vs. deflection; (**b**) absorbed energy of BFRP modified with different contents of star-like polymer.

In a low-velocity impact test, three points on the contact-force vs. deflection graph (Figure 10a) are of prime importance: the first peak signifies initial damage caused by the impactor, the highest peak is the maximum contact force applied, and the third is when the specimen is punctured (if failure occurs). The forces observed at each of these points ascertain the puncture behaviour of the specimens.

**Figure 11:**
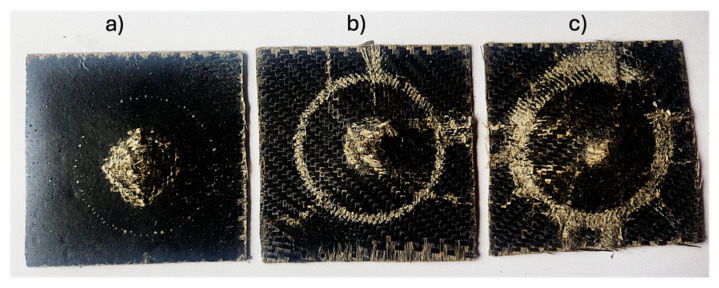
Images of specimens post-impact: (**a**) neat epoxy; (**b**) 0.5 wt.%; (**c**) 1 wt.% of star-like polymer.

In the case of neat epoxy specimens, the impactor had weak resistance to puncture considering there is significant damage at the first drop of the impactor, where the specimen has been majorly damaged, recognized by the steep drop in force required to completely puncture the specimen as seen in Figure 11a. The specimen suffered minimum delamination within the boundary region of impact, thereby showing lower energy-absorption capabilities (Figure 10b). In the case of 0.5 wt.% of star-like polymer-modified BFRP, a higher resistance to puncture in comparison to neat epoxy was observed which reaffirms the reinforcing effect of the polymer. In the contact-force vs. deflection graph, there are two distinct peaks, one where the specimen is first damaged and the second where the maximum force exerted by the impactor punctures the specimen (Figure 10a). It can be seen in Figure 11b that in addition to matrix cracking, there was considerably higher fibre fracture on the non-impacted side, with cracks propagating to the edges with an almost doubling of impact energy absorption. The addition of 1 wt.% shows the maximum resistance to damage, and the contact-force vs. deflection graph shows a distinct peak around 2 kN where the specimen was first damaged (Figure 10a), following which the impactor rebounds on the specimen. In corroboration with the image of the damaged specimens (Figure 11c), the impactor did not puncture the specimen completely, which is why no further distinct peaks are observed in the contact-force vs. deflection graph.

The energy absorbed by the specimen is visualised by the area under the contact force-deflection curve [56]. The specimens were impacted with 50 J of energy of which neat epoxy, 0.5, and 1 wt.% content of star-like polymer composites absorbed 32.4, 63.2, and 96% of the energy, respectively. The greater degree of fracture in the impact boundary region corresponds to greater dissipated energy.

### 3.6. Scanning Electron Microscopy Characterization

**Figure 12:**
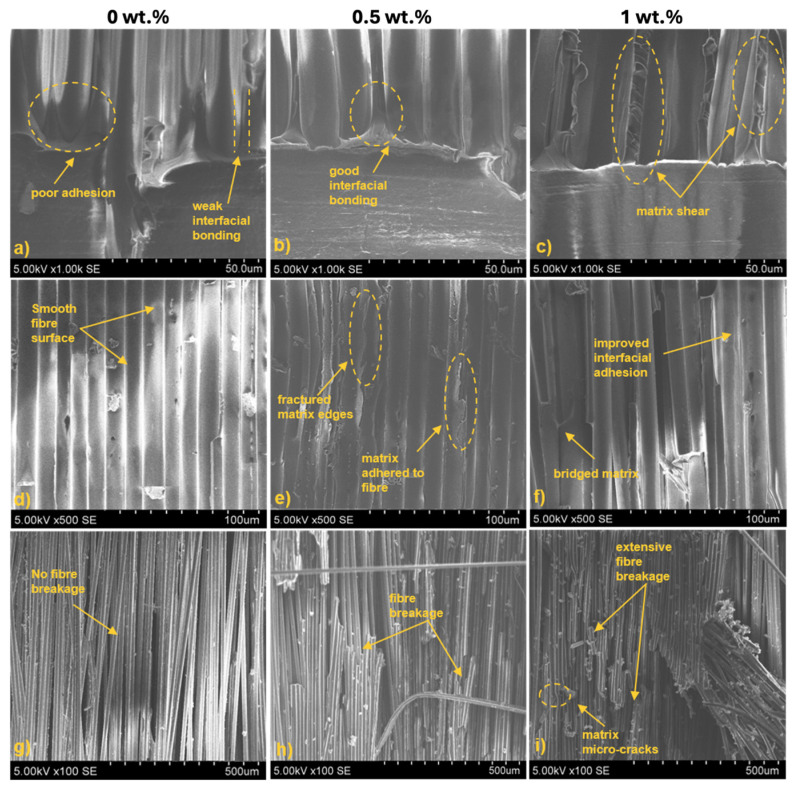
Damage morphology of fractured specimens at different magnifications: (**a**–**c**) at 1000×; (**d**–**f**) 500×; (**g**–**i**) 100× for different contents of star-like polymer.

Fractured specimens from the interlaminar toughness tests were further analysed using SEM. The interface of the delamination initiation was chosen as the area of focus to assess the initiation of matrix breakage, fibre pull-out, fibre bridging, and crack propagation. Figure 12 depicts the damage morphology of the BFRP laminates at different magnifications for neat, 0.5, and 1 wt.%-modified bio-matrix. At 1000× magnification, the interface between the fibre and matrix can be analysed closely. For neat epoxy specimens, clear incohesive zones are visible with gaps present between the fibre and matrix owing to poor interfacial bonding (Figure 12a). With BFRP laminates modified with 0.5 wt.% star-like polymer, an improved interface between the fibre and matrix is observed. No spaces can be discerned (Figure 12b). In BFRP laminates modified with 1 wt.%, excellent adhesion between the fibre and matrix is noted (Figure 12c). Extensive matrix shearing along the fibre depicts elastic deformation of the matrix while maintaining interfacial bonding with the fibre.

At 500×, the interfacial adhesion is visualised over a larger area. In Figure 12d, relatively naked fibre surfaces can be seen with little to no traces of adhered matrix. As we increase the content of star-like polymer as seen in Figure 12e,f, the matrix is adhered over a larger surface of the fibres. In BFRP laminates with 1 wt.% star-like polymer, some matrix bridging can be seen over the surface of two fibres, showing improved interfacial adhesion. At 100×, larger damage modes are visualised, like fibre breakage and cracks in the laminate. For neat epoxy, we can visually detect no damage at the fibre level which indicates poor dispersion of the energy onto the fibres due to weak adhesion at the interlayer (Figure 12g). However, with increasing contents of star-like polymer, more pronounced damage can be observed. Extensive fibre breakage and fractured fibre–matrix edges after crack propagation shows the capabilities of the modified bio-matrix to improve interfacial adhesion by excellent dispersion of energy and thereby resistance to delamination and crack propagation in the laminate.

## 4. Conclusions

In this study, a novel BFRP composite with a high bio-content matrix modified with star-like polymer was developed, demonstrating enhancements in mechanical properties.

The addition of star-like polymer led to a significant increase in the viscosity of the bio-based epoxy. At 0.5 wt.% star-like polymer content, the viscosity was 1.5 times higher than that of the neat epoxy. When the polymer content exceeded 1 wt.%, the viscosity increased even more remarkably. This increase in viscosity may affect the processability of the material, potentially presenting challenges in manufacturing and impacting the overall mechanical performance of the composite.

Although the addition of star-like polymer did not significantly affect the Young’s modulus of the bio-based matrix, the inclusion of 0.25 wt.% star-like polymer led to a 24% increase in ultimate tensile strength compared to neat epoxy.

The incorporation of 0.5 wt.% of star-like polymer resulted in an 8.2 °C increase in the glass transition temperature (from 97.8 °C to 106.0 °C), indicating improved retention in stiffness of the matrix material at higher temperatures.

The interlaminar fracture toughness of the BFRP composite showed a substantial improvement of 45% (from 0.92 kJ/m^2^ to 1.33 kJ/m^2^) with the incorporation of 1 wt.% of star-like polymer, indicating improved resistance to delamination and crack propagation within the laminate.

The most striking result was observed in the low-velocity impact tests, where BFRP laminates modified with 1 wt.% star-like polymer exhibited a threefold increase in energy absorption compared to unmodified laminates. Under the impact of 50 J of energy, composites with 1 wt.% star-like polymer content absorbed 96% of the energy.

These improvements are attributed to the unique branched structure of the star-like polymer, which increases the toughness of the matrix and enhances its ability to resist crack propagation under mechanical stress. It is important to note that the specific content of star-like polymer chosen in this study may not represent the optimal content for all applications. Future studies should focus on tailoring the star-like polymer content for specific performance targets, depending on the application requirements. Nonetheless, this work highlights the potential of using small amounts of star-like polymer to enhance the mechanical properties of BFRP composites with a high bio-content matrix.

## Data Availability

The original contributions presented in the study are included in the article, further inquiries can be directed to the corresponding author.
